# Healthy Eating and Food Hygiene Among Adolescents in Central Kazakhstan: A Cross-Sectional Study

**DOI:** 10.3390/ijerph23080997

**Published:** 2026-07-30

**Authors:** Ulpan Kuandyk, Alua Omarova, Nailya Delellis

**Affiliations:** 1 School of Public Health, Karaganda Medical University, 40 Gogol Street, Karaganda 100008, Kazakhstan; kuandyku@qmu.kz; 2 Department of Scientific Work, Karaganda Medical University, 40 Gogol Street, Karaganda 100008, Kazakhstan; 3 Department of Health Science, The Herbert H. & Grace A. Dow College of Health Professions, Central Michigan University, 1280 E Campus Dr., Mount Pleasant, MI 48859, USA; delel1no@cmich.edu

**Keywords:** feeding behavior, food safety, health education, school food services, schools

## Abstract

**Highlights:**

**Public health relevance—How does this work relate to a public health issue?**

- Healthy eating and food hygiene are important determinants of adolescent health and contribute to the prevention of nutrition-related and infectious diseases.
- Evidence on healthy eating and food hygiene practices among adolescents in Kazakhstan and Central Asia remains limited.

**Public health significance—Why is this work of significance to public health?**

- This study provides a comprehensive assessment of adolescents’ knowledge and practices regarding healthy eating and food hygiene in Central Kazakhstan.
- The study highlights the role of family factors and the school food environment in shaping adolescents’ dietary behaviors.

**Public health implications—What are the key implications or messages for practitioners, policymakers and/or researchers in public health?**

- Strengthening school-based nutrition and food hygiene education may improve adolescents’ dietary habits and health-related behaviors.
- Policies aimed at improving the school food environment and increasing parental involvement may support healthier eating practices among adolescents.

**Abstract:**

Background: Evidence on adolescents’ knowledge and practices related to healthy eating and food hygiene in Kazakhstan remains limited. This study aimed to assess knowledge and behaviors associated with healthy eating and food hygiene among adolescents in Karaganda, Central Kazakhstan. Methods: A cross-sectional study was conducted among 382 students aged 11–18 years from six schools. Data were collected using a bilingual questionnaire and analyzed using Chi-square tests and multivariable logistic regression. Results: Girls were significantly more likely than boys to demonstrate knowledge of healthy eating and food hygiene practices. According to parental reports, 61.5% of adolescents consumed fruit daily, whereas daily fast-food consumption was reported in 7.7% of boys and 7.5% of girls. Food-outsourcing company reports showed that complete meals and drinking water were available in all participating schools; however, dairy products were available in only half of the school cafeterias. Conclusions: The study identified sex- and age-related differences in adolescents’ knowledge and practices regarding healthy eating and food hygiene. The findings highlight the importance of combining nutrition education with improvements in the school food environment and greater parental involvement to promote healthier dietary behaviors among adolescents.

## 1. Introduction

Adolescence is the transition from childhood to adulthood, typically spanning the ages of 10 to 19 years [1]. During this period, eating behaviors are still developing and remain a fundamental component of lifestyle throughout life [2].

Among adolescents, nutritional problems often stem from inadequate nutrition, linked to poor dietary choices. Common patterns include frequent snacking on high-calorie foods, skipping meals (particularly breakfast), irregular eating, high consumption of fast food, and low intake of fruits and vegetables [3,4,5]. In the United States, few high school students meet the recommended intake of fruits and vegetables. Nationally, only 7.1% of high school students met the recommended intake for fruits, whereas only 2% met the recommended intake for vegetables [6].

In many developing countries, adolescents’ nutritional status is further influenced by hygiene-related factors, including lack of clean water, poor sanitation, inadequate hygiene practices, and limited access to toilets [7,8]. Beyond nutrition, insufficient hand hygiene due to a lack of education significantly contributes to global morbidity and mortality [9,10,11,12,13,14,15,16].

Globally, children experience an estimated 1.7 billion cases of diarrhea each year [17], along with approximately 1.5 million deaths from acute respiratory infections. Improper food handling can lead to diarrhea and dysentery, which impair nutrient absorption and negatively affect growth and development [18].

In the context of rising non-communicable diseases and persistent food insecurity, fostering safe eating behaviors among adolescents is particularly relevant to Sustainable Development Goals (SDGs) 2 (Zero Hunger) and 3 (Good Health and Well-Being), which emphasize access to nutritious food and hygiene education for young people [19].

Schools play a central role in creating safe, supportive learning environments that enable students to develop, learn, and grow into healthy, educated citizens [20]. Enhancing the school setting—through teaching basic hygiene skills [21] and monitoring school meals as part of health programs—can substantially contribute to this goal [22,23].

Parents also exert a significant influence beyond the school environment. Evidence shows that parental involvement in nutrition and physical activity programs lowers obesity risk and improves dietary habits in children aged 10–13 years. Joint parental participation increases adolescents’ motivation to adopt healthy behaviors and supports positive long-term outcomes [24].

Beyond family influence, collaboration between schools and food-outsourcing companies in several countries has promoted sustainable dietary improvements and enhanced program effectiveness [25,26]. Systematic reviews further confirm that public–private partnerships in school meals and health reduce obesity risk factors and improve diet quality among children and adolescents [27].

Research by UNICEF and the National Healthy Nutrition Center has shown that sugar and salt consumption in Kazakhstan do not meet international standards and students’ knowledge of healthy eating remains poor [28].

Given the growing challenges associated with adolescent nutrition and food hygiene, promoting healthy and safe eating behaviors among adolescents has become an important public health priority. However, the available studies in Kazakhstan have primarily focused on nutritional status or dietary intake and have rarely examined adolescents’ nutrition-related knowledge, food hygiene practices, parental perspectives, and the school food environment within a single study. Furthermore, evidence from Central Kazakhstan, particularly Karaganda, remains scarce. This lack of comprehensive regional data limits the development of evidence-based school nutrition policies and targeted public health interventions. Therefore, the aim of this study was to comprehensively assess adolescents’ knowledge and behaviors related to healthy eating and food hygiene in Karaganda, Central Kazakhstan, while examining sex- and age-related differences, parental reports on dietary practices, and the characteristics of the school food environment. The findings may support the development of targeted educational and school-based interventions designed to improve nutrition, food hygiene practices, and overall adolescent health.

## 2. Materials and Methods

### 2.1. Study Design and Participants

This cross-sectional survey was conducted in Karaganda, Central Kazakhstan, between September and November 2024 using structured questionnaires. Data were collected from six public schools. Official approval was obtained from the Karaganda Education Department, followed by direct coordination with school principals to arrange school-specific schedules for visits and interviews.

The study population included students in grades 5–11, their parents or legal guardians, and outsourcing companies responsible for school meals. In total, 382 students aged 11–18 years (mean age 14.6 ± 0.21 years) participated. For the comparative analyses, participants were divided into two age groups (11–15 and 16–18 years) according to the educational stages in Kazakhstan. Participants aged 11–15 years generally attend middle school (grades 5–9), whereas those aged 16–18 years attend upper secondary school (grades 10–11). This categorization reflects differences in the educational environment, increasing autonomy, and the development of health-related behaviors during adolescence.

Data were also collected from 382 parents or legal guardians and from six representatives of outsourcing companies.

Students were included if they had sufficient reading and comprehension skills to complete the questionnaire independently within the allotted time. Parents or guardians provided information on adolescents’ home dietary habits and family factors influencing eating behavior. Company representatives reported on the range of products and the organization of school meals, enabling comparison with students’ reported dietary practices.

### 2.2. Inclusion and Exclusion Criteria

Students:

Inclusion criteria: Students in grades 5–11, aged 11–18 years, present on survey days, and providing assent. For minors, written consent from parents or legal guardians was required.

Exclusion criteria: Students younger than 11 or older than 18 years or those who declined participation or those without parental or guardian consent (for participants under 18).

Parents or legal guardians:

Inclusion criteria: Parents or legal guardians of enrolled students who agreed to complete the questionnaire.

Exclusion criteria: Parents or guardians whose children were not participating or who declined participation.

Outsourcing companies responsible for school meals:

Inclusion criteria: Representatives of companies who agreed to participate.

Exclusion criteria: Individuals who declined participation.

### 2.3. Sample Size and Sampling

Karaganda city has 80 schools under the supervision of the Education Department. According to official data for the 2022/2023 academic year, these schools had 72.179 students in grades 1–11. For this study, only students in grades 5–11 (aged 11–18 years) were included, totaling 44.750 students, which served as the reference population (N) for sample size calculation. The sample size was calculated using OpenEpi software (Version 3.01) based on the formula for estimating a population proportion. Assuming a target population of 44.750 students, a 95% confidence level, and a margin of error of 5%, the minimum required sample size was estimated to be 382 students.

Sampling was conducted in two stages: first, six schools were randomly selected from the 80 under the Karaganda Department of Education; second, 382 students from grades 5–11 in these schools were randomly selected using computer-generated sequences. The distribution of the sample by school was as follows: School 1—39 of 476 students; School 2—104 of 1463 students; School 3—91 of 1366 students; School 4—63 of 902 students; School 5—43 of 662 students; and School 6—42 of 811 students.

**Figure 1:**
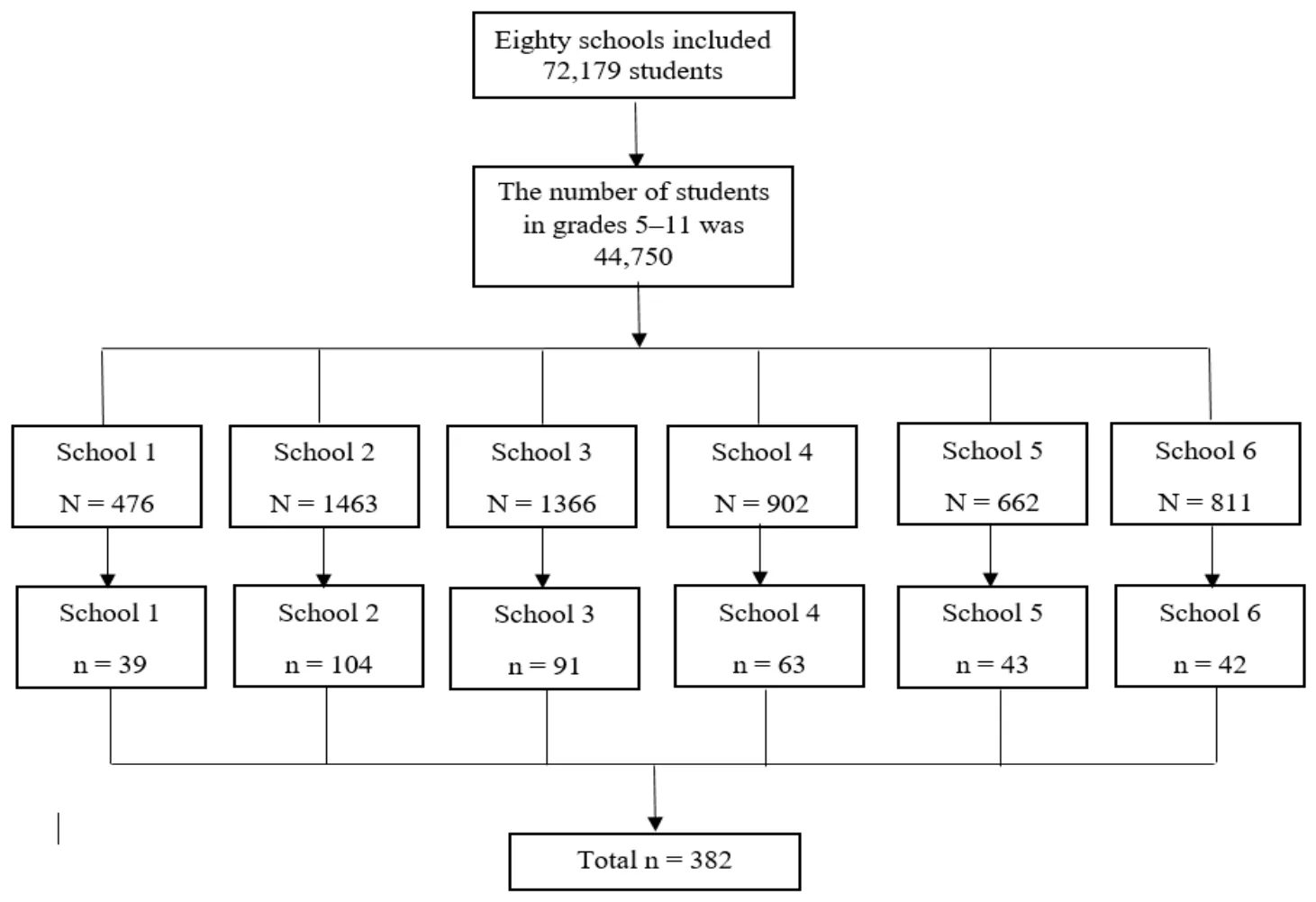
Schematic representation of study participants.

In addition, 382 parents (one per selected student) and 6 representatives of food-outsourcing companies (one per selected school) participated in the study (Figure 1).

### 2.4. Data Collection Tools and Methods

Three online questionnaires were specifically developed for the present study for students, parents or legal guardians, and food-outsourcing companies. The questionnaires were developed by the research team based on a comprehensive review of the scientific literature, the World Health Organization (WHO) recommendations on healthy nutrition and food hygiene [29,30,31,32,33,34] and the current sanitary and epidemiological regulations governing child and adolescent nutrition in the Republic of Kazakhstan [35,36]. The original questionnaires were developed in Russian and subsequently translated into Kazakh using a forward–backward translation procedure to ensure conceptual equivalence between the two language versions. The linguistic validity of the Kazakh version was reviewed by the Language Development Center of Karaganda Medical University. Content validity was assessed by a panel of three public health experts, all holding PhD degrees and having more than 10 years of professional experience in school hygiene and preventive medicine. The experts evaluated the relevance, clarity, and comprehensiveness of each questionnaire item using a four-point Likert scale. Minor editorial revisions were made following expert review. Prior to the main study, pilot testing was conducted among 30 students (15 completed the Kazakh version and 15 the Russian version). The pilot study confirmed the clarity and comprehensibility of the questionnaire, and no substantial modifications were required. All questionnaires were available in both Kazakh and Russian to ensure accessibility and comprehension for all participants.

The student questionnaire assessed:

- Knowledge, attitudes, and practices related to healthy eating and food hygiene.
- Sources of information on nutrition and hygiene.
- The questionnaire included the following sections:

(i) Demographic Data

This section collected information on students’ socioeconomic and educational characteristics, including sex, age, language group, and grade level (5th–11th grades).

(ii) Knowledge

Students’ knowledge of healthy eating and food hygiene was assessed using twelve questions: five yes/no/I don’t know items and seven multiple-choice questions with a single correct answer. The original knowledge section of the questionnaire consisted of 14 items. In the present study, the results for 12 items are reported. Two additional questions regarding the necessity of consuming the entire school lunch and afternoon snack were not presented separately, as they relate to the organization of school meal provision and were not included in the primary scope of the present analysis.

The questions were organized into two domains:

1. Healthy eating—Eight questions that assessed knowledge of:
  ○ The importance of a morning meal (e.g., “Is it necessary to have breakfast in the morning?”)
  ○ The harmful effects of excessive sugar and carbonated drink consumption.
  ○ The recommended daily intake of sugar and salt.
  ○ Daily fluid intake per body weight.
  ○ The optimal interval between breakfast at home and the first meal at school.
  ○ The general principles of healthy eating.

These questions provided a comprehensive understanding of students’ awareness of key components of healthy eating and their role in maintaining overall health.

2. Food Hygiene—Four questions that assessed understanding of:
  ○ Routes of microbial transmission through food and water;
  ○ Necessity of washing fruits and vegetables before consumption;
  ○ Selection of safe drinking water at school and at home.

This structure allowed evaluation of both knowledge of nutritional principles and sanitary practices relevant to adolescent health.

The psychometric properties of the original 14-item knowledge scale were evaluated using exploratory factor analysis. Construct validity was supported by an adequate Kaiser–Meyer–Olkin (KMO) measure of sampling adequacy (KMO = 0.771) and a statistically significant Bartlett’s test of sphericity (χ^2^ = 1381.103, *p* < 0.001), indicating that the data were suitable for factor analysis. The internal consistency was acceptable, with a Cronbach’s alpha coefficient of 0.71, exceeding the commonly accepted threshold of 0.70.

(iii) Practices

This section assessed students’ practical skills in healthy eating and food hygiene, including compliance with hygiene standards related to food intake and handling. Behavioral practices were evaluated using 11 questions, grouped into two domains: healthy eating and food hygiene.

1. Healthy eating—Eight questions that evaluated students’ actual eating behaviors and adherence to healthy eating practices, including:
  ○ Regularity of breakfast (e.g., “Do you have breakfast in the morning?”)
  ○ Frequency of consumption of sugary foods and carbonated drinks.
  ○ Daily intake of sugar and salt.
  ○ Volume of fluids consumed relative to body weight.
  ○ Interval between meals at home and at school.
  ○ Types of foods consumed (e.g., “What type of diet do you adhere to?”)

These questions allowed assessment of students’ compliance with recommended dietary practices. All information on dietary practices, including reported sugar, salt, and fluid intake, was based on participants’ self-reported estimates and did not represent objectively measured dietary intake.

2. Food Hygiene—Three questions that assessed compliance with basic sanitary standards:
  ○ “Do you wash fruits and vegetables before eating?”
  ○ “What kind of water do you drink from the available source at school?”
  ○ “What kind of water do you drink from the available source at home?”

Responses reflected adherence to hygiene practices aimed at preventing foodborne illnesses and infections.

To obtain an overall measure of healthy eating and food hygiene practices, a composite practice index was developed. The index included indicators reflecting meal regularity, participation in school meals, safe food and drinking-water practices, and dietary behaviors related to food and beverage choices. According to Diamantopoulos and Winklhofer [37], formative indices combine conceptually distinct indicators, each representing an independent dimension of the construct rather than reflecting a single underlying latent variable. Therefore, high internal consistency between individual indicators is not expected, and Cronbach’s alpha is not considered an appropriate measure of reliability for formative indices. Accordingly, internal consistency was not assessed for the composite practice index.

Before calculating the composite practice index, responses were recoded so that higher values represented healthier dietary and food hygiene practices. Responses consistent with recommended practices were coded as 1, whereas responses inconsistent with recommendations were coded as 0. The composite practice score was calculated by summing the scores across all 11 practice indicators. The theoretical score ranged from 0 to 11 points, whereas the observed scores in the study sample ranged from 1 to 11 points, with a mean score of 6.61 ± 1.78 points.

(iv) Informational Involvement

This section assessed students’ sources of information on healthy eating and food hygiene. Students were asked whether their parents and teachers provided guidance on the principles of healthy eating and whether they used the information posted on school cafeteria boards (e.g., menu, operating hours, and healthy eating recommendations). These data allowed evaluation of students’ engagement with the educational environment and the accessibility of information on key aspects of healthy eating at school and at home.

The parent/guardian questionnaire focused on adolescents’ diets and adherence to healthy eating principles. It included:

1. Demographic information—Relationship to the child, child’s age, sex, grade, and language of instruction.
2. Dietary preferences and frequency of food consumption—Parents reported how often their children consumed each food group using five categories: daily, often (4–6 days/week), sometimes (1–3 days/week), less than once a week, and never.

Food groups included:

- Porridge and other grain-based dishes, pasta products;
- Beef, horsemeat, and other red meat;
- Poultry (e.g., chicken, turkey);
- Fish and fish-based dishes;
- Milk, kefir, and other fermented dairy drinks;
- Dairy products (e.g., yogurt, cheese);
- Cottage cheese and related dishes (e.g., baked goods, soufflé, cheesecakes);
- Vegetables and fruits;
- Sausages, frankfurters, wieners;
- Eggs and egg-based dishes;
- Fast food (e.g., hamburgers, pizza, shawarma);
- Chips and savory snacks;
- Ketchup, mayonnaise;
- Cakes and pastries;
- Chocolate and chocolate-based items;
- Sugar-sweetened carbonated beverages, sugar-sweetened drinks (e.g., compote, kissel, fruit drinks);
- Drinking water.

The food-outsourcing company questionnaire assessed school meal practices, product assortment, and meal formats, including:

(i) Product assortment: Baked goods (pies, pizza, etc.), sandwiches, hot dogs, chocolate and chocolate-based items, vegetable salads and fresh vegetables, dairy products including drinks, fruit and mixed juices, sugar-sweetened drinks (nectars and fruit drinks), carbonated soft drinks, bottled water, confectionery (cookies, wafers, and glazed sweets), cereal and fruit bars, marshmallows, fruit leather (strips), jelly candies, and fresh fruit;
(ii) Meal organization: Set breakfasts, lunches, and afternoon meals; a “free-choice” system (purchase of individual dishes); alternative set menus; and provision of snacks or tea breaks when full meals were unavailable.

Psychometric evaluation was performed only for the student questionnaire sections assessing nutrition-related knowledge and healthy eating and food hygiene practices because these represented multi-item scales intended to measure latent constructs. The section assessing information sources, as well as the parent/guardian and food-outsourcing company questionnaires, was designed to collect descriptive factual information (e.g., food consumption frequency, sources of nutrition information, and characteristics of the school food environment) rather than latent constructs. Therefore, psychometric evaluation and internal consistency analysis were not considered appropriate for these instruments.

### 2.5. Statistical Analysis

Data were collected using Google Forms. Statistical analyses were performed using IBM SPSS Statistics (version 17.0). Categorical variables were summarized as frequencies and percentages, with 95% confidence intervals (CIs) calculated where appropriate. Variables included sex, age, age groups, and the types and organization of school meals. Pearson’s Chi-square test and Fisher’s exact test (for expected counts < 10) were used to evaluate differences in students’ knowledge and practices regarding healthy eating and food hygiene by sex and age group. For the descriptive analyses, each questionnaire item was analyzed separately; composite knowledge and practice scores were not used. However, for the multivariable logistic regression analysis, a composite nutrition-related knowledge score (0–12 points) was calculated and included as an independent variable. To identify factors independently associated with healthy eating and food hygiene practices, a multivariable binary logistic regression analysis was performed. The dependent variable was the level of healthy eating and food hygiene practices, coded as 0 = low and 1 = high. Sex, age group, and the nutrition-related knowledge score (0–12 points) were included in the model as independent variables. Adjusted odds ratios (ORs) with 95% confidence intervals (95% CIs) were calculated. A significance level of *p* < 0.05 was applied. Given the exploratory nature of the study, no adjustment for multiple comparisons was applied. Therefore, statistically significant findings should be interpreted with appropriate caution.

### 2.6. Ethical Considerations

Ethical approval for this study was obtained from the Local Bioethics Commission at Karaganda Medical University (Kazakhstan) on 6 February 2024 (Protocol No. 9, dated 29 December 2023). Written informed consent was obtained from all participants, their parents or legal guardians, school principals, and representatives responsible for education prior to data collection. Consent forms included the lead researcher’s contact information and a detailed description of the study’s purpose and procedures. Participants were informed that participation was voluntary and that they could withdraw at any time without any consequences. Before the survey, parents were briefed on the questionnaire’s objectives, and school administrators requested their permission for students’ participation.

## 3. Results

### 3.1. Characteristics of Participants

We collected data using structured questionnaires. Students completed a survey assessing their knowledge of healthy eating and dietary habits. Parents provided information on their children’s home diets, while representatives of food-outsourcing companies described the products offered and the organization of school meals. Student results were analyzed by sex (boys and girls) and age group (11–15 years and 16–18 years).

Table 1 presents the main characteristics of the study participants. The study included 382 students (47.4% boys, 52.6% girls) with a mean age of 14.69 ± 0.21 years (range: 11–18). Students were categorized into two age groups: 11–15 years (51.3%) and 16–18 years (48.7%).

A total of 382 parents participated, including 92.9% mothers, 6.3% fathers, and 0.8% grandmothers.

The study also included six representatives of food-outsourcing companies.

### 3.2. Healthy Eating and Food Hygiene Among Adolescents: Knowledge and Behavioral Patterns of Students

Table 2 shows students’ knowledge of healthy eating and basic food hygiene (*n* = 382). Topics included meal routines at school, the effects of sugar and carbonated drinks, handwashing, selection of safe drinking water, and related aspects. *p* < 0.05.

Girls demonstrated higher levels of knowledge than boys for several indicators. A greater proportion of girls correctly identified the harmful effects of excessive consumption of sugary foods (95% vs. 88.4%, *p* < 0.05) and carbonated drinks (97% vs. 89.0%, *p* < 0.05) and recognized the importance of washing fruits and vegetables before consumption (98% vs. 91.2%, *p* < 0.05). Girls were also more likely to identify boiled or dispenser water as the recommended source of drinking water at school (85.6% vs. 75.1%, *p* < 0.05).

Gender differences were also observed for recommended intake levels: sugar (≤25 g/day, 55.2% of girls vs. 40.3% of boys, *p* < 0.05) and salt (≤5 g/day, 48.3% of girls vs. 38.7% of boys, *p* < 0.05).

Statistically significant age differences were observed for certain indicators. Students aged 11–15 years were more likely than those aged 16–18 years to correctly identify the recommended daily salt intake (≤5 g) (50.5% vs. 36.6%, *p* < 0.05). Conversely, a greater proportion of older adolescents reported not knowing the recommended daily salt intake (36.6% vs. 27.6%, *p* < 0.05). A substantial proportion of respondents were unable to identify the recommended daily fluid intake, with a higher percentage observed among adolescents aged 16–18 years than among those aged 11–15 years (49.5% vs. 41.3%, *p* < 0.05).

Table 3 presents students’ practices related to healthy eating and basic food hygiene, including regularity of breakfast, frequency of sweets and carbonated drink consumption, handling of fruits and vegetables, choice of drinking water at school and home, daily intake of sugar, salt, and fluids, intervals between meals, and predominant dietary patterns.

Analysis by sex revealed several significant differences. Boys reported having regular breakfast more often than girls (86.7% vs. 76.6%, *p* < 0.05). For home drinking water, girls preferred boiled water or water from a dispenser more frequently than boys (89.6% vs. 80.1%, *p* < 0.05). A higher proportion of girls reported consuming no more than 25 g of sugar per day (70.1% vs. 55.8%, *p* < 0.05) and reported consuming no more than 5 g of salt per day (67.7% vs. 49.7%, *p* < 0.05). Conversely, boys more frequently reported consuming 1.0–1.5 L of fluids per day than girls (40.3% vs. 26.4%, *p* < 0.05).

Age-related differences were also observed: students aged 11–15 years consumed sugary products more frequently than those aged 16–18 years (53.6% vs. 40.3%, *p* < 0.05).

Table 4 presents adolescents’ awareness of healthy eating and their use of the information posted in the school cafeteria. Girls more often reported being told about the principles of healthy eating at home compared to boys (92% vs. 84%, *p* < 0.05). Approximately 70% of students indicated that they received information about healthy eating at school (during lessons), with no significant differences by gender (*p* = 0.443) or age group (*p* = 0.120). Over 60% of students in both groups found the information boards in the school cafeteria useful, with slightly higher proportions among girls (61.2% vs. 55.2% of boys) and students aged 11–15 years (62.8% vs. 53.8% of those aged 16–18 years). These differences were not statistically significant (*p* = 0.277 and *p* = 0.134, respectively).

#### Independent Predictors of Healthy Eating and Food Hygiene Practices

To identify factors independently associated with healthy eating and food hygiene practices, a multivariable logistic regression analysis was performed (Table 5). After adjustment for age group and knowledge score, girls had significantly higher odds of demonstrating healthy eating and food hygiene practices than boys (adjusted OR = 1.59; 95% CI: 1.01–2.49; *p* = 0.043). No statistically significant association was observed between age group and healthy eating and food hygiene practices (adjusted OR = 0.86; 95% CI: 0.55–1.34; *p* = 0.499). Each one-point increase in the knowledge score was independently associated with a 60% increase in the odds of exhibiting healthy eating and food hygiene practices (adjusted OR = 1.60; 95% CI: 1.40–1.83; *p* < 0.001).

**Figure 2:**
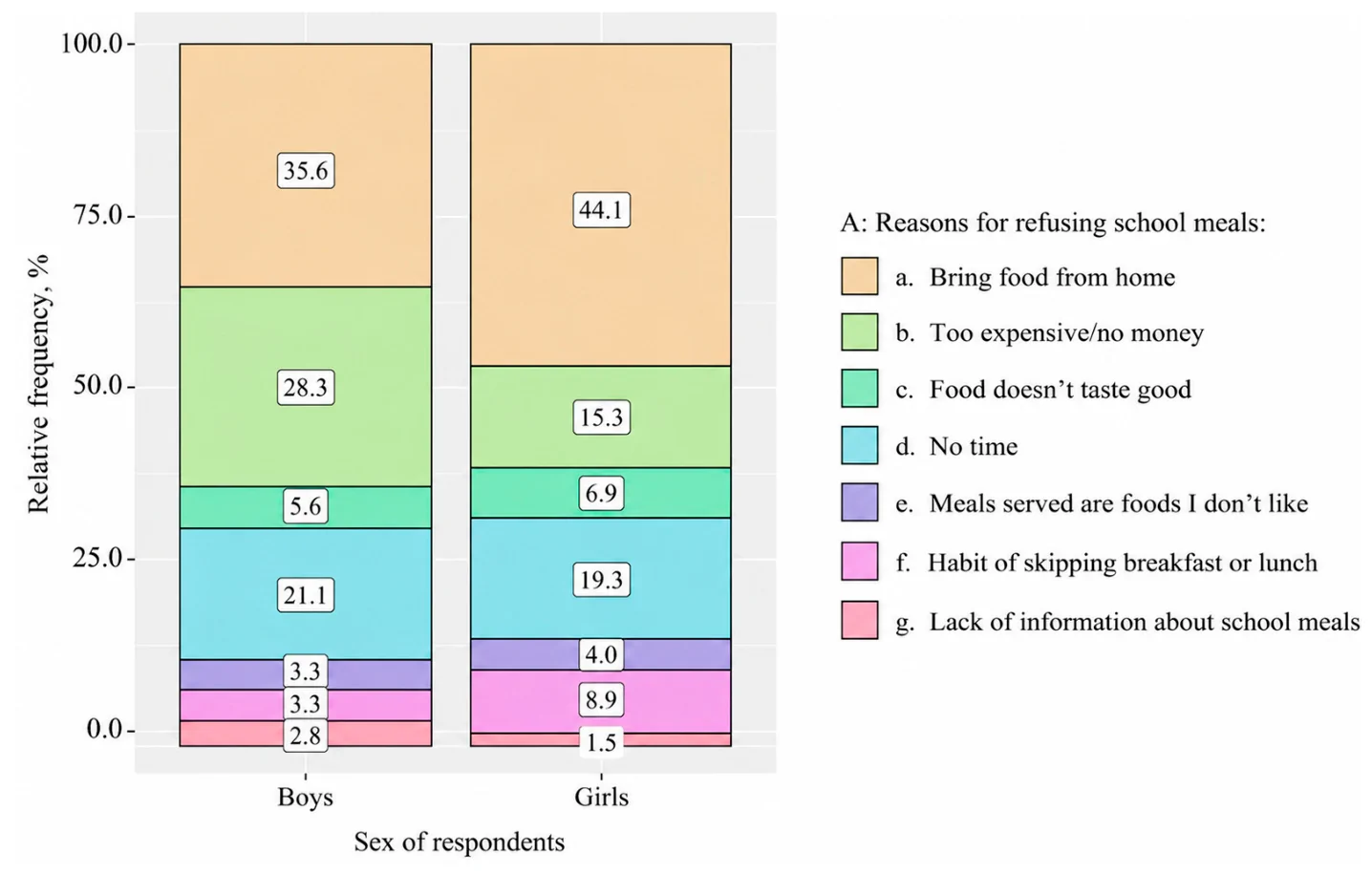

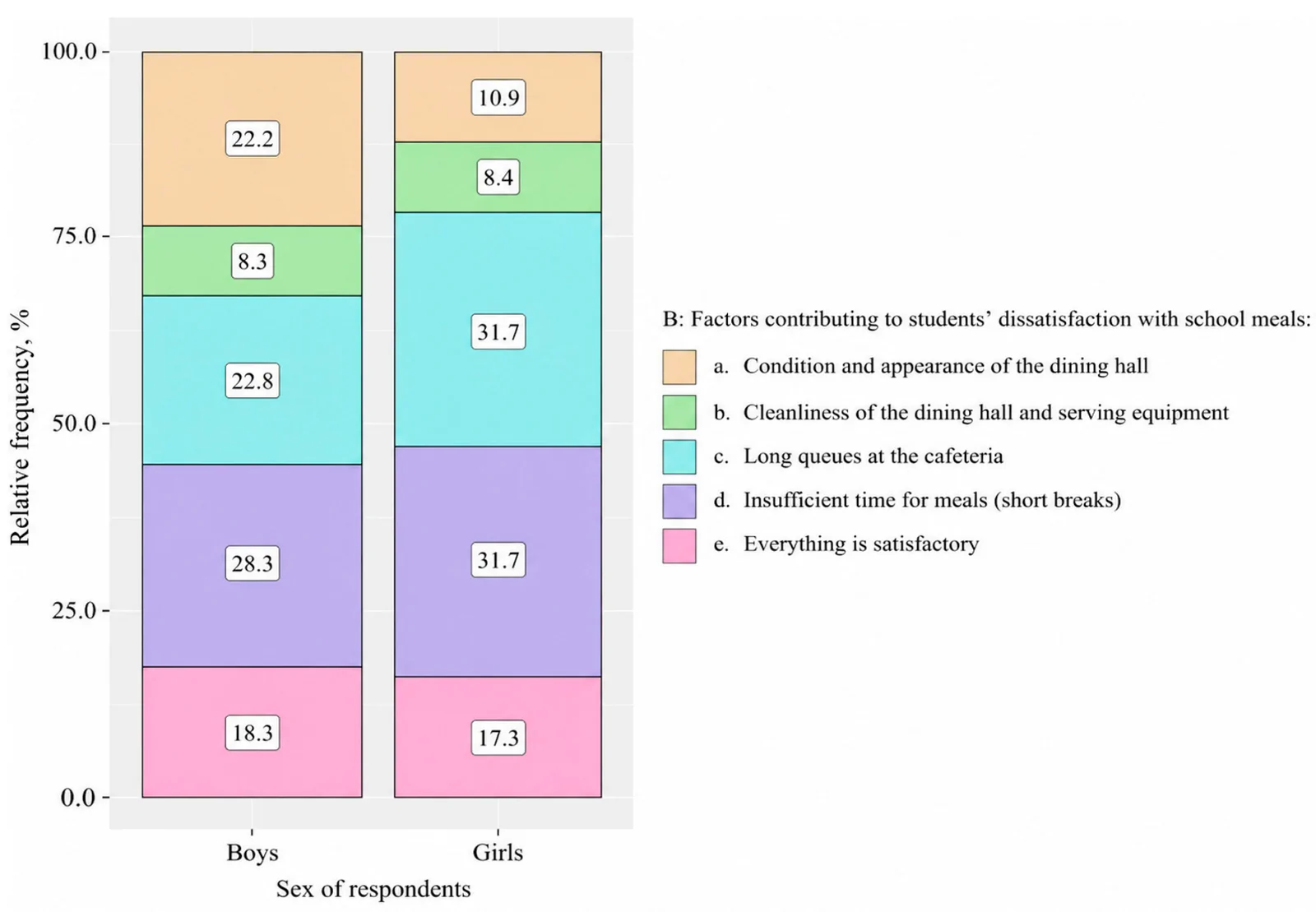
Adolescents’ perception of the school canteen: conditions and factors of dissatisfaction.

Figure 2A shows reasons for refusing school meals by sex. The most common reason for both boys (35.6%) and girls (44.1%) was bringing food from home. Boys reported lack of money more frequently than girls (28.3% vs. 15.3%), while lack of time was reported by 21.1% of boys and 19.3% of girls.

Figure 2B presents factors contributing to dissatisfaction with school meal organization. The main concerns in both sex groups were short breaks preventing a full meal (31.7% of girls vs. 28.3% of boys) and long queues (31.7% of girls vs. 22.8% of boys). The design and condition of the dining hall caused more dissatisfaction among boys (22.2%) than girls (10.9%). Around one in five students reported no issues (18.3% of boys vs. 17.3% of girls). Other minor concerns included the dining hall design and the sanitary conditions of the premises.

### 3.3. Analysis of Student Diets Based on Parent or Legal Guardian Reports

Table 6 presents the frequency of adolescents’ food consumption according to parental reports. Approximately 61.5% of adolescents consumed fruit daily. Among meat products, beef and horsemeat were the most commonly consumed, with approximately half of adolescents consuming them daily. Poultry was also regularly included in adolescents’ diets. Foods with low nutritional value, including fast food, chips, sweetened carbonated beverages, and chocolate, were widely consumed. Daily fast-food consumption was reported in 7.7% of boys and 7.5% of girls. Daily consumption of chips was reported in 9.9% of boys and 8.5% of girls, while daily consumption of sweetened carbonated beverages was reported in 12.2% of boys and 10.9% of girls. Daily drinking-water consumption was reported by 84% of boys and 86.6% of girls.

### 3.4. Analysis of School Meals from Outsourcing Company Reports

Table 7 presents the product range and meal formats in school cafeterias operating under an outsourced model. All cafeterias offered baked goods, vegetable salads, bottled water, and fresh fruit. Students could select a full set meal (breakfast, lunch, or afternoon snack) or individual dishes/alternative menus. Dairy products were available in half of the cafeterias (50%, 95% CI: 11.8–88.2) and natural juices in 66.7%. Sweetened carbonated beverages were not offered in any cafeteria, while drinks with added sugar were available in only one (16.7%, 95% CI: 0.4–64.1). Snacks with added sugar, including chocolate products, butter cookies, glazed cookies, waffles, marshmallows, fruit leather, fruit jelly and cereal or fruit-cereal bars, were not sold. Most cafeterias (83.3%, 95% CI: 35.9–99.6) provided quick snacks or tea when full meals were unavailable.

## 4. Discussion

The study findings indicate that adolescents demonstrated good awareness of the harmful effects of sugary foods, carbonated drinks, and basic food hygiene practices. However, important knowledge gaps were identified regarding the recommended daily intake of water, sugar, and salt. In addition, clear sex- and age-related differences were observed in both nutrition-related knowledge and dietary behavior. These findings align with previous research showing that adolescent girls often adopt a more responsible approach to diet and food hygiene, including water source selection and adherence to sugar and salt intake recommendations [38,39,40,41,42,43]. Specifically, girls demonstrated greater knowledge of recommended salt and sugar consumption, were more likely to choose boiled or filtered water, and reported healthier practices overall. One possible explanation for the higher level of nutrition-related knowledge among girls is greater health consciousness and stronger social norms regarding body weight and healthy eating, as suggested in previous studies. This interpretation is consistent with the findings of Deslippe et al., who reported that exogenous motivators (social approval and weight control) were more influential among girls, whereas endogenous motivators (pleasure and productivity) were more influential among boys [39]. Interestingly, in our study, 86.7% of boys and 76.6% of girls regularly ate breakfast, which contrasts with some reports suggesting girls maintain more stable eating patterns [44]. This discrepancy may reflect culturally specific factors that warrant further regional investigation.

Although the assessment of food hygiene in the present study primarily focused on indicators such as washing fruits and vegetables before consumption and the choice of drinking-water source, these measures may serve as proxy indicators of adherence to food hygiene practices. Washing fresh fruits and vegetables before consumption helps reduce the risk of contamination with pathogenic microorganisms, parasites, pesticide residues, and other contaminants, while the use of safe drinking water is one of the key measures for preventing foodborne and waterborne diseases. According to the WHO, the use of safe drinking water, adherence to personal and food hygiene practices, and washing fruits and vegetables before consumption are essential measures for the prevention of foodborne diseases [45]. Therefore, the indicators used in the present study reflect important aspects of adolescents’ preventive behaviors related to reducing the risk of foodborne diseases and have important public health implications.

The findings are particularly relevant in the context of Kazakhstan, where national assessments have reported excessive consumption of sugar and salt among schoolchildren and insufficient adherence to healthy eating recommendations [28]. The observed differences between knowledge and reported dietary practices suggest that higher nutritional awareness does not always correspond to healthier reported dietary practices [46]. These findings highlight the importance of strengthening both nutrition education and the school food environment, while also encouraging greater parental involvement in shaping healthy eating habits among adolescents.

According to the Health Belief Model, high awareness of healthy eating does not necessarily translate into corresponding behavior, as the adoption of sustainable habits depends on perceived threats, barriers, cues, and the subjective value of actions [47,48].

One possible explanation, suggested by previous European studies, is that older adolescents may become more independent in their eating behaviors and pay less attention to nutrition-related information [49]. These factors were not directly assessed in the present study.

Analysis of the school environment indicated that students evaluated cafeteria information boards positively but applied the knowledge in practice to a limited extent: over 60% reported receiving information during lessons, yet fewer applied it daily. This supports the notion that theory alone, without a supportive environment, is insufficient, consistent with the Health Belief Model and socio-ecological framework [47,49].

Another important finding was that bringing food from home was the most frequently reported reason for refusing school meals. Although the present study did not assess the types of foods brought from home or purchased outside school, this finding suggests that some adolescents may rely on foods brought from home or obtained outside the school food service environment. This may partly explain the frequent consumption of fast food, chocolate, chips, and sweetened beverages observed in the present study despite the absence or limited availability of these products in school cafeterias. Further research is needed to identify the main sources of these foods.

According to parental reports, adolescents’ home diets substantially complemented school meals. Parents reported frequent consumption of fast food and sweetened beverages and relatively low daily consumption of vegetables and dairy products. These findings align with global school-based student health surveys, which indicate that adolescents in many countries rarely consume vegetables and dairy products but regularly include fast food and sugary drinks [50].

According to parental reports, girls tended to consume fruits and dairy products more frequently than boys and older adolescents tended to report more regular intake of protein and dairy products. However, these differences were not statistically significant and should therefore be interpreted with caution. These findings are consistent with previous research suggesting that the family environment may play an important role in adolescents’ dietary habits and can reinforce or weaken school-based nutrition interventions [51]. According to the socio-ecological model, the family acts as an important microsystem influencing adolescents’ eating behaviors [49].

Data from food-outsourcing companies indicated that school meal organization generally met basic standards: all cafeterias offered complete meals, water, and fruits, with no sweetened carbonated beverages. However, only half of the cafeterias provided dairy products, and the availability of healthy snacks was limited. While the absence of chocolate bars and confectionery is positive, the restricted range of healthier options (e.g., cereal bars and yogurts) reduces the potential of school meals to support chronic disease prevention.

Nevertheless, parent reports indicated that adolescents frequently consumed chocolate, chips, and sweetened beverages. This finding likely reflects food consumed or purchased outside the school food service environment, as the dietary data represented adolescents’ overall food intake, whereas the assessment of outsourcing companies was limited to foods available in school cafeterias. These findings suggest that improving school meals alone may not be sufficient to promote healthy eating behaviors and should be complemented by interventions targeting the home and community food environment [26,52]. 

The present study highlights the potential role of the school food environment in adolescents’ dietary behaviors. Although all participating schools provided complete meals and drinking water, the limited availability of dairy products and healthy snack options may restrict students’ opportunities to translate nutrition knowledge into healthier food choices. This observation supports growing evidence that nutrition education alone is unlikely to be sufficient insufficient unless supported by an enabling food environment [53].

These findings are consistent with European studies showing that, even when sweetened beverages and confectionery are restricted, the availability of healthy snacks remains limited, reducing the effectiveness of school-based healthy-eating programs [29,54]. This underscores the need for closer collaboration between schools and food-outsourcing companies and the establishment of standardized product offerings.

The findings may have practical implications for multiple stakeholders. Based on the observed dietary patterns and school food environment, teachers and school administrators could consider expanding the variety of healthy foods, improving meal conditions, and strengthening nutrition education while taking age- and sex-related differences into account. Parents should actively engage in educational activities to enhance adolescents’ receptiveness to nutrition information. Food-outsourcing companies could consider increasing the availability of dairy products and healthy snack options and improving the consistency of school meal offerings. These findings may help inform public health authorities in the development of school nutrition policies and strategies to strengthen intersectoral collaboration for improving adolescent nutrition.

This study not only confirms sex- and age-related differences in adolescents’ knowledge and dietary behaviors but also highlights the complex factors influencing the development of healthy habits. The findings suggest that information alone is insufficient and call for a systemic approach, including adaptation of educational strategies, enhancement of school meal infrastructure, and active parental involvement. Interventions should account for the social, cultural, and infrastructural context of Kazakhstan to ensure feasibility and effectiveness.

Study limitations: This study has several limitations. First, it was conducted in only six schools in Karaganda, which limits the generalizability of the findings to the broader region or the country. In addition, information on the school food environment was obtained from representatives of only six food-outsourcing companies (one per participating school). Therefore, these findings should be interpreted with caution and should not be considered representative of all schools or catering providers in Kazakhstan. Second, dietary intake was assessed using parent-reported data, while information on knowledge and dietary practices was based on students’ self-reports. Consequently, these data may be subject to recall bias and social desirability bias. Parents may have unintentionally overestimated the frequency of healthy food consumption, such as daily fruit and vegetable intake, or underestimated the consumption of less healthy foods, including fast food and sugar-sweetened beverages. Therefore, these findings should be interpreted with appropriate caution. Third, although the questionnaire underwent content validation, pilot testing, and psychometric evaluation, it was specifically developed for this study. Additional validation in larger and independent populations would further strengthen its applicability in different settings. Fourth, multiple statistical comparisons were performed without adjustment for multiple testing. Therefore, some statistically significant findings may represent chance associations and should be interpreted with caution. Finally, the cross-sectional study design does not allow causal relationships to be established between knowledge, dietary practices, and school environment factors. Future research should include larger and more geographically diverse samples, additional validation of the questionnaire in independent populations, and longitudinal or intervention-based designs to strengthen the evidence.

## 5. Conclusions

Adolescents in Karaganda demonstrated good awareness of the harmful effects of sugary foods, carbonated drinks, and basic food hygiene practices. However, important knowledge gaps were identified regarding the recommended daily intake of water, sugar, and salt, indicating that knowledge of specific dietary recommendations remains insufficient. The study identified sex- and age-related differences in nutrition-related knowledge and self-reported dietary practices. Differences were also observed in parent-reported dietary patterns and characteristics of the school food environment. These findings provide updated evidence on adolescents’ nutrition-related knowledge, dietary practices, and food hygiene in Central Kazakhstan and may support future research in this field.

## Figures and Tables

**Table 1:** Sociodemographic profile of participants (*n* = 382).

Indicators	Frequency (*n*)	Percentage (%)	95% CI
Boys	181	47.4	42.3–52.5
Girls	201	52.6	47.5–57.7
Total students	382	100	88.1–100
Age group			
11–15 years	196	51.3	46.2–56.4
16–18 years	186	48.7	43.6–53.8
Relationship to student			
Mother	355	92.9	89.9–95.3
Father	24	6.3	4.1–9.2
Grandmothers	3	0.8	0.2–2.3

**Table 2:** Knowledge of healthy eating and food hygiene among adolescent students by sex and age (*n* = 382).

Knowledge of Healthy Eating and Food Hygiene	Sex		*p*	Age		*p*
	Boys, *n* (%)	Girls, *n* (%)		11–15 Years, *n* (%)	16–18 Years, *n* (%)	
Healthy eating						
Is it necessary to have breakfast in the morning?			0.746			0.895
Yes **	164 (90.6)	181 (90)		177 (90.3)	168 (90.3)	
No	8 (4.4)	7 (3.5)		7 (3.6)	8 (4.3)	
I don’t know	9 (5)	13 (6.5)		12 (6.1)	10 (5.4)	
Are you aware of the harmful effects of sugary foods?			0.023 *			0.569
Yes **	160 (88.4)	191 (95)		178 (90.8)	173 (93)	
No	11 (6.1)	8 (4)		12 (6.1)	7 (3.8)	
I don’t know	10 (5.5)	2 (1)		6 (3.1)	6 (3.2)	
Are you aware of the harmful effects of carbonated drinks?			0.005 *			0.533
Yes **	161 (89)	195 (97)		180 (91.8)	176 (94.6)	
No	8 (4.4)	4 (2)		7 (3.6)	5 (2.7)	
I don’t know	12 (6.6)	2 (1)		9 (4.6)	5 (2.7)	
What is the maximum recommended daily sugar intake?			0.017 *			0.493
≤25 g (about 2 tablespoons or 6 teaspoons) **	73 (40.3)	111 (55.2)		101 (51.5)	83 (44.6)	
≤50 g (about 4 tablespoons or 12 teaspoons)	37 (20.4)	27 (13.4)		34 (17.3)	30 (16.1)	
≤75 g (about 6 tablespoons or 18 teaspoons)	6 (3.3)	5 (2.5)		4 (2)	7 (3.8)	
≤100 g (about 8 tablespoons or 24 teaspoons)	8 (4.4)	2 (1)		4 (2)	6 (3.2)	
I don’t know	57 (31.5)	56 (27.9)		53 (27)	60 (32.3)	
What is the maximum recommended daily salt intake?			0.035 *			0.019 *
≤5 g (slightly less than 1 teaspoon) **	70 (38.7)	97 (48.3)		99 (50.5)	68 (36.6)	
≤10 g (about 2 teaspoons)	30 (16.6)	29 (14.4)		32 (16.3)	27 (14.5)	
≤15 g (about 3 teaspoons)	12 (6.6)	9 (4.5)		7 (3.6)	14 (7.5)	
≤20 g (about 4 teaspoons)	11 (6.1)	2 (1)		4 (2)	9 (4.8)	
I don’t know	58 (32)	64 (31.8)		54 (27.6)	68 (36.6)	
What is the recommended daily intake of water (fluids)?			0.560			0.028 *
Less than 0.5 L	15 (8.3)	14 (7)		22 (11.2)	7 (3.8)	
0.5–1.0 L	21 (11.6)	27 (13.4)		27 (13.8)	21 (11.3)	
1.0–1.5 L	36 (19.9)	28 (13.9)		36 (18.4)	28 (15.1)	
More than 1.5 L	30 (16.6)	38 (18.9)		30 (15.3)	38 (20.4)	
I don’t know	79 (43.6)	94 (46.8)		81 (41.3)	92 (49.5)	
What is the interval between your breakfast at home and your first meal at school (or vice versa)?			0.756			0.271
<2 h	37 (20.4)	37 (18.4)		45 (23)	29 (15.6)	
2–3 h	57 (31.5)	71 (35.3)		69 (35.2)	59 (31.7)	
3–4 h **	41 (22.7)	44 (21.9)		42 (21.4)	43 (23.1)	
4–6 h	11 (6.1)	18 (9)		12 (6.1)	17 (9.1)	
>6 h	3 (1.7)	2 (1)		2 (1)	3 (1.6)	
I don’t know	32 (17.7)	29 (14.4)		26 (13.3)	35 (18.8)	
What does it mean to eat healthily?			0.078			0.775
Consuming nutrients in sufficient amounts and combinations **	149 (82.3)	175 (87.1)		164 (83.7)	160 (86)	
Eating fast food (e.g., burgers, sandwiches, pizza, fries, chips, sushi)	5 (2.8)	2 (1)		3 (1.5)	4 (2.2)	
Following a mono diet (one food group only)	5 (2.8)	1 (0.5)		3 (1.5)	3 (1.6)	
Eating many sweets	3 (1.7)	0 (0)		1 (0.5)	2 (1.1)	
I don’t know	19 (10.5)	23 (11.4)		25 (12.8)	17 (9.1)	
Food hygiene						
Can germs be transmitted through food and water?			0.057			0.354
Yes **	155 (85.6)	165 (82.1)		159 (81.1)	161 (86.6)	
No	10 (5.5)	5 (2.5)		9 (4.6)	6 (3.2)	
I don’t know	16 (8.8)	31 (15.4)		28 (14.3)	19 (10.2)	
Should fruits and vegetables be washed before eating?			0.009 *			0.943
Yes **	165 (91.2)	197 (98)		185 (94.4)	177 (95.2)	
No	8 (4.4)	1 (0.5)		5 (2.6)	4 (2.2)	
I don’t know	8 (4.4)	3 (1.5)		6 (3.1)	5 (2.7)	
What type of water should be consumed at school?			0.010 *			0.549
Boiled and/or dispenser water **	136 (75.1)	172 (85.6)		153 (78.1)	155 (83.3)	
Tap water	12 (6.6)	5 (2.5)		11 (5.6)	6 (3.2)	
Well or borehole water	10 (5.5)	2 (1)		7 (3.6)	5 (2.7)	
I don’t know	23 (12.7)	22 (10.9)		25 (12.8)	20 (10.8)	
What type of water should be consumed at home?			0.102			0.297
Boiled and/or dispenser water **	144 (79.6)	175 (87.1)		158 (80.6)	161 (86.6)	
Tap water	17 (9.4)	7 (3.5)		14 (7.1)	10 (5.4)	
Well or borehole water	10 (5.5)	10 (5)		14 (7.1)	6 (3.2)	
I don’t know	10 (5.5)	9 (4.5)		10 (5.1)	9 (4.8)	

Note. * Pearson’s Chi-square test indicated a statistically significant difference (*p* < 0.05). ** The correct answer.

**Table 3:** Practices of healthy eating and food hygiene among adolescent students by sex and age (*n* = 382).

Healthy Eating and Food Hygiene Practices	Sex		*p*	Age		*p*
	Boys, *n* (%)	Girls, *n* (%)		11–15 Years, *n* (%)	16–18 Years, *n* (%)	
Healthy eating						
Do you have breakfast in the morning?			0.011 *			0.051
Yes	157 (86.7)	154 (76.6)		167 (85.2)	144 (77.4)	
No	24 (13.3)	47 (23.4)		29 (14.8)	42 (22.6)	
How often do you eat sweets?			0.363			0.002 *
Every day	67 (37)	61 (30.3)		47 (24)	81 (43.5)	
2–3 times a week	76 (42)	104 (51.7)		105 (53.6)	75 (40.3)	
Once a week	25 (13.8)	23 (11.4)		30 (15.3)	18 (9.7)	
Once a month	6 (3.3)	4 (2)		5 (2.6)	5 (2.7)	
Never	7 (3.9)	9 (4.5)		9 (4.6)	7 (3.8)	
How often do you drink carbonated drinks?			0.063			0.244
Every day	25 (13.8)	13 (6.5)		17 (8.7)	21 (11.3)	
2–3 times a week	63 (34.8)	69 (34.3)		62 (31.6)	70 (37.6)	
Once a week	54 (29.8)	56 (27.9)		58 (29.6)	52 (28)	
Once a month	23 (12.7)	40 (19.9)		33 (16.8)	30 (16.1)	
Never	16 (8.8)	23 (11.4)		26 (13.3)	13 (7)	
How many grams of sugar do you consume per day?			0.013 *			0.236
≤25 g	101 (55.8)	141 (70.1)		134 (68.4)	108 (58.1)	
≤50 g	48 (26.5)	44 (21.9)		41 (20.9)	51 (27.4)	
≤75 g	16 (8.8)	11 (5.5)		11 (5.6)	16 (8.6)	
≤100 g	15 (8.3)	5 (2.5)		10 (5.1)	10 (5.4)	
None	1 (0.6)	0 (0)		0 (0)	1 (0.5)	
How many grams of salt do you consume per day?			0.001 *			0.193
≤5 g.	90 (49.7)	136 (67.7)		120 (61.2)	106 (57)	
≤10 g.	53 (29.3)	54 (26.9)		56 (28.6)	51 (27.4)	
≤15 g.	22 (12.2)	5 (2.5)		14 (7.1)	13 (7)	
≤20 g.	14 (7.7)	6 (3)		6 (3.1)	14 (7.5)	
None	2 (1.1)	0 (0)		0 (0)	2 (1.1)	
How much water (fluid) do you consume per day?			0.004 *			0.236
Less than 0.5 L	33 (18.2)	38 (18.9)		42 (21.4)	29 (15.6)	
0.5–1.0 L	35 (19.3)	68 (33.8)		53 (27)	50 (26.9)	
1.0–1.5 L	73 (40.3)	53 (26.4)		66 (33.7)	60 (32.3)	
More than 1.5 L	40 (22.1)	42 (20.9)		35 (17.9)	47 (25.3)	
What is the interval between breakfast and the first meal at school or at home?			0.094			0.525
<2 h	47 (26)	33 (16.4)		44 (22.4)	36 (19.4)	
2–3 h	43 (23.8)	70 (34.8)		58 (29.6)	55 (29.6)	
3–4 h	39 (21.5)	42 (20.9)		43 (21.9)	38 (20.4)	
4–6 h	15 (8.3)	21 (10.4)		13 (6.6)	23 (12.4)	
>6 h	6 (3.3)	4 (2)		6 (3.1)	4 (2.2)	
I don’t know	31 (17.1)	31 (15.4)		32 (16.3)	30 (16.1)	
What type of diet do you usually follow?			0.151			0.709
Balanced diet (grains, dairy, meat, vegetables, fruits)	141 (77.9)	176 (87.6)		162 (82.7)	155 (83.3)	
High in confectionery	15 (8.3)	10 (5)		11 (5.6)	14 (7.5)	
Instant foods (e.g., noodles, mashed potatoes)	11 (6.1)	8 (4)		12 (6.1)	7 (3.8)	
Fast food (e.g., hamburgers, pizza, fries, chips, sushi)	9 (5)	4 (2)		6 (3.1)	7 (3.8)	
Processed foods (e.g., canned goods, sausages)	5 (2.8)	3 (1.5)		5 (2.6)	3 (1.6)	
Food hygiene						
How often do you wash fruits and vegetables before eating?			0.092			0.053
Always	153 (84.5)	185 (92)		177 (90.3)	161 (86.6)	
Often	15 (8.3)	10 (5)		12 (6.1)	13 (7)	
Sometimes	9 (5)	2 (1)		4 (2)	7 (3.8)	
Rarely	2 (1.1)	3 (1.5)		0 (0)	5 (2.7)	
Never	2 (1.1)	1 (0.5)		3 (1.5)	0 (0)	
What type of water do you usually drink at school?			0.522			0.227
Boiled and/or dispenser water	148 (82.7)	166 (84.3)		160 (82.5)	154 (84.6)	
Tap water	22 (12.3)	18 (9.1)		25 (12.9)	15 (8.2)	
I don’t know	9 (5)	13 (6.6)		9 (4.6)	13 (7.1)	
What type of water do you usually drink at home?			0.011 *			0.181
Boiled and/or dispenser water	145 (80.1)	180 (89.6)		166 (84.7)	159 (85.5)	
Tap water	23 (12.7)	8 (4)		18 (9.2)	13 (7)	
Well or borehole water	12 (6.6)	10 (5)		12 (6.1)	10 (5.4)	
I don’t know	1 (0.6)	3 (1.5)		0 (0)	4 (2.2)	

Note. * The Chi-square test was considered significant at *p* < 0.05.

**Table 4:** Awareness of healthy eating among adolescents by sex and age group (*n* = 382).

Awareness of Healthy Eating Among Adolescents	Sex		*p*	Age		*p*
	Boys, *n* (%)	Girls, *n* (%)		11–15 Years, *n* (%)	16–18 Years, *n* (%)	
Do your parents talk to you about healthy eating in relation to school meals?			0.443			0.120
Yes	119 (65.7)	143 (71.1)		143 (73)	119 (64)	
No	43 (23.8)	43 (21.4)		40 (20.4)	46 (24.7)	
I don’t know	19 (10.5)	15 (7.5)		13 (6.6)	21 (11.3)	
Do your parents talk to you about the principles of healthy eating at home?			0.036 *			0.657
Yes	152 (84)	185 (92)		175 (89.3)	162 (87.1)	
No	18 (9.9)	12 (6)		13 (6.6)	17 (9.1)	
I don’t know	11 (6.1)	4 (2)		13 (6.6)	17 (9.1)	
Do you use the information board in the school cafeteria (menu, opening hours, dining rules, healthy eating information)?			0.277			0.134
Yes, it is important	99 (55)	124 (61.4)		123 (62.8)	100 (53.8)	
No, I’m not interested	80 (44.4)	78 (38.6)		73 (37.2)	85 (45.7)	
I don’t know	1 (0.6)	0 (0)		0 (0)	1 (0.5)	

Note. * Chi-square test results were considered significant at *p* < 0.05.

**Table 5:** Multivariable logistic regression analysis of factors associated with healthy eating and food hygiene practices among adolescents.

Variable	β	Adjusted OR	95% CI	*p* Value
Male	Reference	1.00	–	–
Female	0.463	1.59	1.01–2.49	0.043
Age group				
11–15 years	Reference	1.00	–	–
16–18 years	−0.155	0.86	0.55–1.34	0.499
Knowledge score (per 1-point increase)	0.470	1.60	1.40–1.83	<0.001

OR = odds ratio; CI = confidence interval.

**Table 6:** Parental report of adolescents’ food consumption frequency by sex and age (*n* = 382).

Product and Frequency	Sex		*p*	Age		*p*
	Boys, *n* (%)	Girls, *n* (%)		11–15 Years, *n* (%)	16–18 Years, *n* (%)	
Porridges and cereals			0.418			0.375
Every day	34 (18.8)	37 (18.4)		30 (15.3)	41 (22)	
Often (4–6 days a week)	43 (23.8)	44 (21.9)		47 (24.0)	40 (21.5)	
Sometimes (1–3 days a week)	66 (36.5)	88 (43.8)		84 (42.9)	70 (37.6)	
Less than once a week	13 (7.2)	15 (7.5)		16 (8.2)	12 (6.5)	
Never	25 (13.8)	17 (8.5)		19 (9.7)	23 (12.4)	
Pasta			0.475			0.510
Every day	22 (12.2)	17 (8.5)		16 (8.2)	23 (12.4)	
Often (4–6 days a week)	62 (34.3)	60 (29.9)		64 (32.7)	58 (31.2)	
Sometimes (1–3 days a week)	76 (42)	94 (46.8)		89 (45.4)	81 (43.5)	
Less than once a week	16 (8.8)	25 (12.4)		20 (10.2)	21 (11.3)	
Never	5 (2.8)	5 (2.5)		7 (3.6)	3 (1.6)	
Beef, horsemeat, etc.			0.559			0.458
Every day	93 (51.4)	90 (44.8)		89 (45.4)	94 (50.5)	
Often (4–6 days a week)	57 (31.5)	63 (31.3)		70 (35.7)	50 (26.9)	
Sometimes (1–3 days a week)	21 (11.6)	32 (15.9)		25 (12.8)	28 (15.1)	
Less than once a week	6 (3.3)	10 (5)		7 (3.6)	9 (4.8)	
Never	4 (2.2)	6 (3)		5 (2.6)	5 (2.7)	
Poultry meat: chicken, turkey, etc.			0.513			0.211
Every day	46 (25.4)	58 (28.9)		46 (23.5)	58 (31.2)	
Often (4–6 days a week)	70 (38.7)	73 (36.3)		82 (41.8)	61 (32.8)	
Sometimes (1–3 days a week)	48 (26.5)	53 (26.4)		48 (24.5)	53 (28.5)	
Less than once a week	11 (6.1)	15 (7.5)		16 (8.2)	10 (5.4)	
Never	6 (3.3)	2 (1)		4 (2)	4 (2.2)	
Fish and fish dishes			0.987			0.636
Every day	14 (7.7)	13 (6.5)		11 (5.6)	16 (8.6)	
Often (4–6 days a week)	36 (19.9)	42 (20.9)		40 (20.4)	38 (20.4)	
Sometimes (1–3 days a week)	70 (38.7)	78 (38.8)		76 (38.8)	72 (38.7)	
Less than once a week	38 (21)	44 (21.9)		41 (20.9)	41 (22)	
Never	23 (12.7)	24 (11.9)		28 (14.3)	19 (10.2)	
Milk, kefir, fermented milk products, etc.			0.657			0.643
Every day	72 (39.8)	78 (38.8)		82 (41.8)	68 (36.6)	
Often (4–6 days a week)	57 (31.5)	57 (28.4)		57 (29.1)	57 (30.6)	
Sometimes (1–3 days a week)	35 (19.3)	37 (18.4)		32 (16.3)	40 (21.5)	
Less than once a week	10 (5.5)	18 (9)		16 (8.2)	12 (6.5)	
Never	7 (3.9)	11 (5.5)		9 (4.6)	9 (4.8)	
Dairy products (yogurt, cheese, etc.)			0.385			0.130
Every day	67 (37)	81 (40.3)		73 (37.2)	75 (40.3)	
Often (4–6 days a week)	65 (35.9)	67 (33.3)		70 (35.7)	62 (33.3)	
Sometimes (1–3 days a week)	34 (18.8)	29 (14.4)		36 (18.4)	27 (14.5)	
Less than once a week	10 (5.5)	20 (10)		16 (8.2)	14 (7.5)	
Never	5 (2.8)	4 (2)		1 (0.5)	8 (4.3)	
Cottage cheese dishes with it (baked foods, soufflé, cheeses, etc.)			0.433			0.156
Every day	43 (23.8)	45 (22.4)		35 (17.9)	53 (28.5)	
Often (4–6 days a week)	60 (33.1)	68 (33.8)		72 (36.7)	56 (30.1)	
Sometimes (1–3 days a week)	51 (28.2)	50 (24.9)		56 (28.6)	45 (24.2)	
Less than once a week	14 (7.7)	27 (13.4)		21 (10.7)	20 (10.8)	
Never	13 (7.2)	11 (5.5)		12 (6.1)	12 (6.5)	
Vegetables			0.126			0.862
Every day	90 (49.7)	125 (62.2)		114 (58.2)	101 (54.3)	
Often (4–6 days a week)	58 (32)	46 (22.9)		49 (25)	55 (29.6)	
Sometimes (1–3 days a week)	22 (12.2)	23 (11.4)		24 (12.2)	21 (11.3)	
Less than once a week	5 (2.8)	4 (2)		4 (2)	5 (2.7)	
Never	6 (3.3)	3 (1.5)		5 (2.6)	4 (2.2)	
Potatoes			0.779			0.412
Every day	44 (24.3)	47 (23.4)		43 (21.9)	48 (25.8)	
Often (4–6 days a week)	83 (45.9)	82 (40.8)		88 (44.9)	77 (41.4)	
Sometimes (1–3 days a week)	43 (23.8)	58 (28.9)		54 (27.6)	47 (25.3)	
Less than once a week	8 (4.4)	11 (5.5)		10 (5.1)	9 (4.8)	
Never	3 (1.7)	3 (1.5)		1 (0.5)	5 (2.7)	
Fruits			0.113			0.275
Every day	102 (56.4)	133 (66.2)		119 (60.7)	116 (62.4)	
Often (4–6 days a week)	54 (29.8)	48 (23.9)		56 (28.6)	46 (24.7)	
Sometimes (1–3 days a week)	21 (11.6)	12 (6)		14 (7.1)	19 (10.2)	
Less than once a week	3 (1.7)	7 (3.5)		7 (3.6)	3 (1.6)	
Never	1 (0.6)	1 (0.5)		0 (0)	2 (1.1)	
Sausages, knackwursts, frankfurters			0.348			0.392
Every day	38 (21)	41 (20.4)		36 (18.4)	43 (23.1)	
Often (4–6 days a week)	55 (30.4)	52 (25.9)		52 (26.5)	55 (29.6)	
Sometimes (1–3 days a week)	52 (28.7)	74 (36.8)		70 (35.7)	56 (30.1)	
Less than once a week	22 (12.2)	16 (8)		18 (9.2)	20 (10.8)	
Never	14 (7.7)	18 (9)		20 (10.2)	12 (6.5)	
Eggs and egg dishes			0.404			0.374
Every day	49 (27.1)	60 (29.9)		58 (29.6)	51 (27.4)	
Often (4–6 days a week)	76 (42)	71 (35.3)		76 (38.8)	71 (38.2)	
Sometimes (1–3 days a week)	34 (18.8)	51 (25.4)		46 (23.5)	39 (21)	
Less than once a week	15 (8.3)	14 (7)		13 (6.6)	16 (8.6)	
Never	7 (3.9)	5 (2.5)		3 (1.5)	9 (4.8)	
Fast food (hamburgers, pizza, shawarma, etc.)			0.738			0.366
Every day	14 (7.7)	15 (7.5)		10 (5.1)	19 (10.2)	
Often (4–6 days a week)	36 (19.9)	33 (16.4)		34 (17.3)	35 (18.8)	
Sometimes (1–3 days a week)	60 (33.1)	76 (37.8)		73 (37.2)	63 (33.9)	
Less than once a week	54 (29.8)	63 (31.3)		61 (31.1)	56 (30.1)	
Never	17 (9.4)	14 (7)		18 (9.2)	13 (7)	
Chips, croutons			0.454			0.336
Every day	18 (9.9)	17 (8.5)		14 (7.1)	21 (11.3)	
Often (4–6 days a week)	37 (20.4)	32 (15.9)		34 (17.3)	35 (18.8)	
Sometimes (1–3 days a week)	53 (29.3)	71 (35.3)		68 (34.7)	56 (30.1)	
Less than once a week	47 (26)	59 (29.4)		51 (26)	55 (29.6)	
Never	26 (14.4)	22 (10.9)		29 (14.8)	19 (10.2)	
Ketchup, mayonnaise			0.943			0.898
Every day	15 (8.3)	21 (10.4)		18 (9.2)	18 (9.7)	
Often (4–6 days a week)	38 (21)	42 (20.9)		41 (20.9)	39 (21)	
Sometimes (1–3 days a week)	64 (35.4)	66 (32.8)		69 (35.2)	61 (32.8)	
Less than once a week	40 (22.1)	47 (23.4)		41 (20.9)	46 (24.7)	
Never	24 (13.3)	25 (12.4)		27 (13.8)	22 (11.8)	
Cakes and pastries			0.915			0.767
Every day	16 (8.8)	15 (7.5)		13 (6.6)	18 (9.7)	
Often (4–6 days a week)	36 (19.9)	47 (23.4)		41 (20.9)	42 (22.6)	
Sometimes (1–3 days a week)	68 (37.6)	75 (37.3)		78 (39.8)	65 (34.9)	
Less than once a week	50 (27.6)	51 (25.4)		52 (26.5)	49 (26.3)	
Never	11 (6.1)	13 (6.5)		12 (6.1)	12 (6.5)	
Chocolate			0.992			0.780
Every day	31 (17.1)	35 (17.4)		31 (15.8)	35 (18.8)	
Often (4–6 days a week)	57 (31.5)	62 (30.8)		62 (31.6)	57 (30.6)	
Sometimes (1–3 days a week)	58 (32)	66 (32.8)		67 (34.2)	57 (30.6)	
Less than once a week	28 (15.5)	32 (15.9)		31 (15.8)	29 (15.6)	
Never	7 (3.9)	6 (3)		5 (2.6)	8 (4.3)	
Sweet carbonated drinks			0.989			0.691
Every day	22 (12.2)	22 (10.9)		23 (11.7)	21 (11.3)	
Often (4–6 days a week)	48 (26.5)	54 (26.9)		51 (26)	51 (27.4)	
Sometimes (1–3 days a week)	58 (32)	62 (30.8)		65 (33.2)	55 (29.6)	
Less than once a week	35 (19.3)	42 (20.9)		41 (20.9)	36 (19.4)	
Never	18 (9.9)	21 (10.4)		16 (8.2)	23 (12.4)	
Drinks with added sugar (compote, kissel, fruit drink, etc.)			0.948			0.677
Every day	23 (12.7)	25 (12.4)		22 (11.2)	26 (14)	
Often (4–6 days a week)	58 (32)	62 (30.8)		67 (34.2)	53 (28.5)	
Sometimes (1–3 days a week)	60 (33.1)	69 (34.3)		65 (33.2)	64 (34.4)	
Less than once a week	29 (16)	29 (14.4)		27 (13.8)	31 (16.7)	
Never	11 (6.1)	16 (8)		15 (7.7)	12 (6.5)	
Drinking water			0.898			0.170
Every day	152 (84)	174 (86.6)		168 (85.7)	158 (84.9)	
Often (4–6 days a week)	18 (9.9)	17 (8.5)		22 (11.2)	13 (7)	
Sometimes (1–3 days a week)	6 (3.3)	7 (3.5)		4 (2)	9 (4.8)	
Less than once a week	2 (1.1)	1 (0.5)		1 (0.5)	2 (1.1)	
Never	3 (1.7)	2 (1)		1 (0.5)	4 (2.2)	

**Table 7:** Range and organization of school meals according to school reports (*n* = 6).

Meals	Categories	Abs. (%)	95% CI
Baked foods (pies, pizza, etc.)	Yes	6 (100)	54.1–100
Sandwiches, hot dogs	No	6 (100)	54.1–100
Chocolate and chocolate products	No	6 (100)	54.1–100
Vegetable salads, fresh vegetables	Yes	6 (100)	54.1–100
Dairy products, including drinks	Yes	3 (50)	11.8–88.2
	No	3 (50)	11.8–88.2
Fruit and vegetable juices	Yes	4 (66.7)	22.3–95.7
	No	2 (33.3)	4.3–77.7
Sweetened juice drinks (nectars, etc.)	Yes	1 (16.7)	0.4–64.1
	No	5 (83.3)	35.9–99.6
Sweet carbonated drinks	No	6 (100)	54.1–100
Bottled drinking water	Yes	6 (100)	54.1–100
Butter cookies, glazed cookies, waffles	No	6 (100)	54.1–100
Cereal and fruit-cereal bars	No	6 (100)	54.1–100
Marshmallows, fruit leather, fruit jelly	No	6 (100)	54.1–100
Fresh fruits	Yes	6 (100)	54.1–100
Set meals (breakfast, lunch, afternoon snack)	Yes	6 (100)	54.1–100
“Free choice”—purchase individual dishes	Yes	6 (100)	54.1–100
Alternative set menus per meal	Yes	6 (100)	54.1–100
Snacks/tea only when full meals are unavailable	Yes	5 (83.3)	35.9–99.6
	No	1 (16.7)	0.4–64.1

Note. 95% CI = 95% Confidence Interval.

## Data Availability

The datasets generated and analyzed during the current study are not publicly available due to ethical and privacy restrictions but may be made available from the corresponding author on reasonable request subject to approval by the relevant ethics committee.
